# Effect of Free Fatty Acids on Inflammatory Gene Expression and Hydrogen Peroxide Production by Ex Vivo Blood Mononuclear Cells

**DOI:** 10.3390/nu12010146

**Published:** 2020-01-04

**Authors:** Antoni Sureda, Miquel Martorell, Maria del Mar Bibiloni, Cristina Bouzas, Laura Gallardo-Alfaro, David Mateos, Xavier Capó, Josep A. Tur, Antoni Pons

**Affiliations:** 1 Research Group on Community Nutrition and Oxidative Stress, University of Balearic Islands & IDISBA, 07122 Palma de Mallorca, Spain; antoni.sureda@uib.es (A.S.); mar.bibiloni@uib.es (M.d.M.B.); cristinabouvel@gmail.com (C.B.); lauragala3@gmail.com (L.G.-A.); david-mateos@hotmail.es (D.M.); xavier.capo@uib.es (X.C.); antonipons@uib.es (A.P.); 2 CIBEROBN (Physiopathology of Obesity and Nutrition), Instituto de Salud Carlos III, 28029 Madrid, Spain; 3 IDISBA (Institut d’Investigació Sanitària de les Illes Balears), Fundació Institut d’Investigació Sanitària de les Illes Balears, 07120 Palma de Mallorca, Spain; 4 Department of Nutrition and Dietetics, Faculty of Pharmacy, University of Concepcion, 4070386 Concepcion, Chile; martorellpons@gmail.com

**Keywords:** PBMC, fatty acids, LPS, gene expression, ROS production

## Abstract

The aim of this study was to assess free fatty acids’ (FAs) *ex vivo* anti-/proinflammatory capabilities and their influence on inflammatory gene expression and H_2_O_2_ production by human peripheral blood mononuclear cells (PBMCs). Anthropometric and clinical measurements were performed in 26 participants with metabolic syndrome. Isolated PBMCs were incubated *ex vivo* for 2 h with several free fatty acids—palmitic, oleic, α-linolenic, γ-linolenic, arachidonic and docosahexaenoic at 50 μM, and lipopolysaccharide (LPS) alone or in combination. H_2_O_2_ production and IL6, NFκB, TLR2, TNFα, and COX-2 gene expressions were determined. Palmitic, γ-linolenic, and arachidonic acids showed minor effects on inflammatory gene expression, whereas oleic, α-linolenic, and docosahexaenoic acids reduced proinflammatory gene expression in LPS-stimulated PBMCs. Arachidonic and α-linolenic acids treatment enhanced LPS-stimulated H_2_O_2_ production by PBMCs, while palmitic, oleic, γ-linolenic, and docosahexaenoic acids did not exert significant effects. Oleic, α-linolenic, and docosahexaenoic acids induced anti-inflammatory responses in PBMCs. Arachidonic and α-linolenic acids enhanced the oxidative status of LPS-stimulated PBMCs. In conclusion, PBMC *ex vivo* assays are useful to assess the anti-/proinflammatory and redox-modulatory effects of fatty acids or other food bioactive compounds.

## 1. Introduction

The field of bioactive food compounds and their effectiveness on human health is gaining interest. The effects of these compounds are mainly assessed using animal models, but in vivo and *ex vivo* studies are also useful methodologies. Peripheral blood mononuclear cells (PBMCs), largely composed of lymphocytes and monocytes, are an easily obtainable fraction of blood cells with high research possibilities to test the effects of food compounds [1,2]. These cells are a promising target tissue as changes in their environment are reflected at the level of gene expression [2,3]. PBMCs also express nuclear receptors such as peroxisome proliferator activated receptors (PPAR) [4], which mediate fatty acid (FAs) effects on lipid metabolism, and Toll-like receptors (TLRs) [5], which modulate the immune response though gene expression regulation. Previous reports showed the utility of PBMCs in evidencing the effects of a functional food, training, and acute exercise on mitochondrial oxidative balance, mitochondrial biosynthesis and dynamics, and antioxidant capabilities [6,7].

Dietary FAs alter the FA composition of plasma and cell membranes, including those of immune cells [8,9]. Changes in the FA composition of immune cells or plasma could affect the inflammatory response [6,10] and the ability to produce cytokines and lipid mediators [11]. Cytokine synthesis in immune cells depends on nuclear factor kappa B (NFκB), which is modulated by TLRs [11,12,13]. The activation of TLRs by microbial products or endogenous damage-associated molecular patterns (DAMP) triggers a cascade of intracellular signaling pathways in innate immune cells, inducing an inflammatory response [14]. Lipopolysaccharides (LPS) as major components of the outer membrane of Gram-negative bacteria interact with TLR4, inducing PBMCs immune response and activating the NFκB and cytokine production—interleukin 6 and 8 (IL6, IL8) and tumor necrosis factor alpha (TNFα)—[10,15,16]. It has been suggested that docosahexaenoic acid (DHA) alters immune cell membrane lipid microdomain properties, inhibiting the action of TLRs [12,17]. In this sense, dietary supplementation with omega-3 FAs has been reported to ameliorate the synthesis of IL1b, IL1a, and TNFα in LPS-stimulated PBMCs [10,18]. The immunomodulatory effects of omega-3 FAs on LPS-activated PBMCs have been proposed to be mediated through the regulation of NFκB and PPAR pathways [10,14,19,20]. The PBMC-based human in vitro system has been developed as a model to evaluate the efficacy of several treatments with polyunsaturated fatty acids (PUFAs) on inflammation induced by LPS stimulation [21]. These models are based on the evaluation of the capability of PUFAs to inhibit the enhanced expression of key inflammatory genes or T-cell activation after PBMCs stimulation [22].

The activation and the increased expression of TLRs in obesity and metabolic syndrome are considered a part of the chronic proinflammatory process present in these pathologies [23]. Persistent low-grade inflammation associated with the increase in systemic levels of proinflammatory cytokines favors PBMCs being in a state of preactivation against an immune stimulus [24]. In addition, because the anti-inflammatory effects of omega-3 FAs seem to be related to the capability to inhibit the stimulation of TLRs and the NFκB pathway, we hypothesized that other free FAs could also modulate the expression of anti-/proinflammatory genes and the production of ROS. The aim was to assess the effects of equimolecular concentrations of selected saturated, monounsaturated, and polyunsaturated fatty acids on the expression of inflammatory genes and hydrogen peroxide production by *ex vivo* human PBMCs from patients with metabolic syndrome stimulated with or without LPS stimulation. The fatty acids selected were saturated FA as palmitic acid (C16:0), monounsaturated FA as oleic acid (C18:1n9), polyunsaturated omega 3 FA as α-linolenic acid (C18:3n3) and docosahexaenoic acid (C22:6n3), and polyunsaturated FAs omega 6 as γ-linolenic acid (C18:3n6) and arachidonic acid (C20:4n6). In addition, we proposed a more rapid model for study the inflammatory effects of fatty acids in *ex vivo* human immune cells, adequate for assessing functional properties of functional foods.

## 2. Methods

### 2.1. Experimental Procedure

The study was carried out on 26 inhabitants (55–80 years old) of the Balearic Islands with metabolic syndrome. All subjects volunteered to participate in the study and gave their written informed consent after an explanation of the experimental procedures and before the beginning of the study. The study was conducted according to the guidelines laid down in the Declaration of Helsinki, and all procedures were approved by the Balearic Islands Ethics Committee (approval reference number n° IB/2251/14 PI).

### 2.2. Anthropometric and Clinical Measurements

Anthropometric determinations were carried out by expert dietitians who performed identical and meticulous training to minimize the possible effects of interobserver variation. Height was calculated with a stadiometer (Kawe 44444, Kirchner and Wilhelm GmBH Co., KG, Asperg, Germany), with the head of the patient in the Frankfurt plane. Body weight was measured with a digital scale (Tefal, sc9210, Groupe SEB, Rumilly, FranceTefal). All subjects were weighed barefoot, wearing only their dressed in underwear. The body mass index (BMI, kg/m^2^) was calculated using the measures of weight and height. Waist and hip circumference (cm) were measured to the nearest 0.1 cm using a nonstretch measuring tape (Kawe 43972, Kirchner and Wilhelm GmbH Co., KG, Asperg, Germany), midway between the rib cage and the iliac crest, and as the maximum circumference around the buttocks posteriorly and the symphysis pubis anteriorly. Blood pressure was taken in triplicate in a seated position after 5 min of resting, using a validated semiautomatic oscillometer (Omron HEM-705CP, Hoofddorp, The Netherlands), waiting 5 min between each reading. 

Venous blood samples were acquired from the antecubital vein of participants at 8:00 a.m., after 12 h of overnight fasting, in vacutainers containing EDTA as anticoagulant. Glucose, total cholesterol, HDL cholesterol and triglycerides were determined in an autoanalyzer (Technicon DAX System, Technicon Instruments Corp., Tarrytown, NY, USA). Whereas LDL cholesterol was calculated by the Friedewald formula. Hematological parameters were determined using clinical routine protocols in an automatic flow-cytometer analyzer Technicon H_2_ (Bayer, Leverkusen, Germany) VCS system. 

### 2.3. PBMCs Purification

PBMCs were isolated from blood using an adaptation of the procedure developed by Boyum [17,25], using Ficoll^®^ Paque PLUS (GE Healthcare, Chalfont St Giles, United Kingdom). Blood samples were cautiously introduced on Ficoll (1.5:1 v:v) and were centrifuged at 900 g, for 30 min at 4 °C. The PBMCs layer was carefully collected and transferred to a new tube. The PBMCs fraction was washed twice with PBS and centrifuged for 10 min at 900 g, 4 °C. 

### 2.4. PBMCs Incubation with FA and/or LPS

Incubation of PBMCs in the presence of different FAs and/or the bacterial stimuli LPS were performed in RPMI 1640 culture media containing 2 mM L-glutamine (Sigma–Aldrich, Madrid, Spain). FAs studied were palmitic (C16:0), oleic (C18:1n9), γ-linolenic (C18:3n6), α-linolenic (C18:3n3), arachidonic (C20:4n6), and docosahexaenoic (C22:6n3) acids at 50 μM concentration (Sigma–Aldrich, Madrid, Spain). The activation of PBMCs by addition of bacterial stimuli LPS from *Escherichia coli* (Sigma–Aldrich, Spain) was performed at 1 μg/mL.

PBMCs isolated from 2 mL of whole blood (3320 ± 180 cells/µL) were suspended with 2 mL of RPMI 1640 culture media and were treated as four groups: Control (LPS−, FA−), FA (LPS−, FA+), LPS (LPS+, FA−), and FA plus LPS (LPS+, FA+). The samples were incubated in polypropylene tubes at 37 °C for 2 h with gentle shaking. Aliquots of cell suspension (2 aliquots of 50 μL) were used for H_2_O_2_ production measurement at the beginning of the incubation. Then, another two aliquots of cell suspension (2 aliquots of 50 μL) were used for H_2_O_2_ production measurement at the end of the cells’ incubation. The rest of the cells were pelleted by centrifugation (900 g, 5 min, 4 °C) and were lysed with Tripure reagent (Roche Diagnostics, Mannheim, Germany). 

### 2.5. RNA Extraction and Real-Time PCR Assay

IL6, NFκB, TLR2, TNFα, and cyclooxygenase 2 (COX2) mRNA levels were measured in PBMCs using a multiplex real-time PCR with human 18S rRNA as a reference. Total RNA was purified from cells by Tripure extraction (Roche Diagnostics, Mannheim, Germany). RNA (1 μg) from each subject was reverse-transcribed using Expand Reverse Transcriptase (50 U) (Roche Diagnostics, Mannheim, Germany) and 20 pmol oligo (dT) for 60 min at 37 °C. The obtained cDNA (2.5 μL) was amplified using the Light-Cycler FastStart DNA MasterPLUS SYBR Green I kit (Roche Diagnostics, Mannheim, Germany). The primers used and the amplification conditions are shown in Table 1. The relative quantification was done considering 2^(−ΔΔCt)^ and referring to the values of the control group as 1. 

### 2.6. Hydrogen Peroxide Production

H_2_O_2_ production was measured before and after PBMCs incubation for 2 h in duplicate after immune stimulation with LPS using 2,7-dichlorofluorescin-diacetate (DCFH-DA, Sigma–Aldrich, Madrid, Spain) as an indicator [7]. In a 96-well microplate, 50 μL of PBMCs suspension (before and after incubation for 2 h) of every sample, 50 μL of DCFH-DA (30 μg/mL), and 50 μL LPS (1 μg/mL) in PBS was added. The fluorescence (Ex, 480 nm; Em, 530 nm) was recorded at 37 °C for 1 h in FL 9800 Microplate Fluorescence Reader (Bio-Tek Instruments, Inc., Winooski, VT, USA).

### 2.7. Statistical Analysis

Statistical Package for Social Sciences (IBM SPSS Statistics v.24 for Windows) was used to carry out statistics. Results were shown as mean ± SEM and *p* < 0.05 was considered statistically significant. The normal distribution and homogeneity of the data were assessed by Kolmogorov–Smirnov and Levene tests, respectively. The statistical significance for ROS production and anthropometric and clinical measurements was assessed by a paired sample *t*-test. The statistical significance of the data about gene expression was assessed by two-way analysis of variance (ANOVA). The statistical factors analyzed were FA and LPS treatments. The data sets with significant differences were further analyzed by the one-way ANOVA test with LSD post hoc test in order to determine the differences between the different groups.

## 3. Results

Anthropometric, hematological, and biochemical characteristics of participants in the study are shown in Table 2. The participants were obese subjects (BMI higher than 30), hyperglycemic (blood glucose higher than 110 mg/dL), and hypertriglyceridemic (blood triglyceride higher than 150 mg/mL) with blood cell count values and cellular characteristics within the normal clinical range of the general population. All participants harbored the metabolic syndrome.

**Figure 1:**
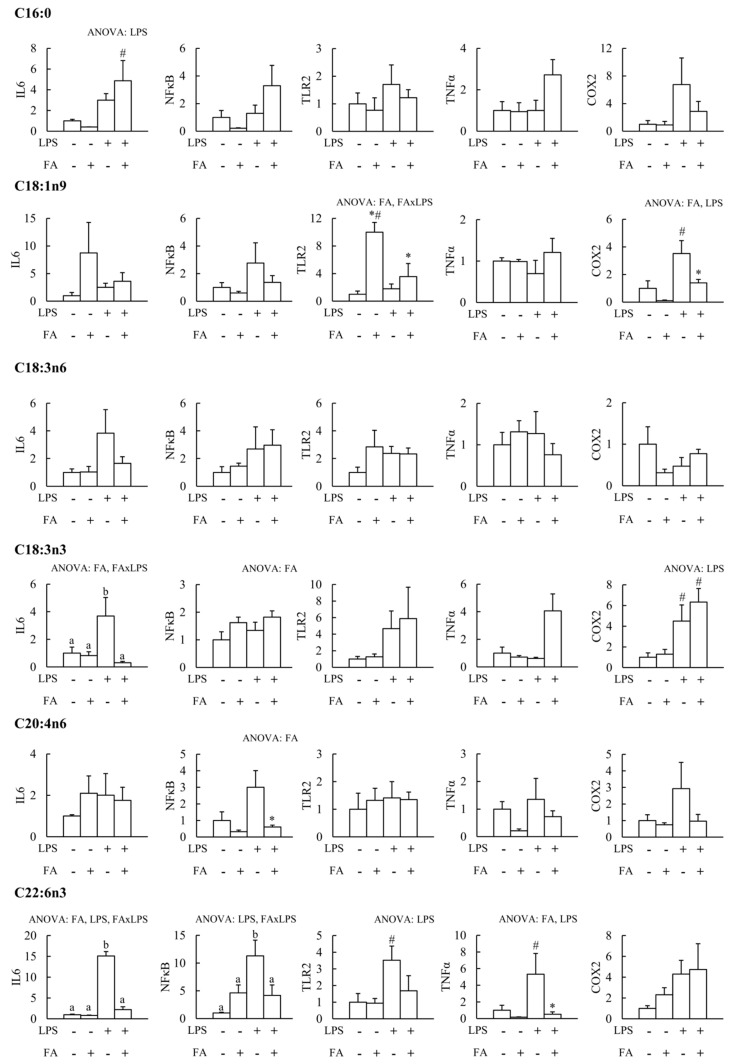
Effect of fatty acids (FA) and lipopolysaccharide (LPS) on peripheral blood mononuclear cells’ (PBMCs) anti-/proinflammatory gene expression. ANOVA: FA, LPS, or FA/LPS indicates significant differences (p < 0.05) by effect of fatty acids (FA), lipopolysaccharide (LPS), or interaction (FA/LPS) by two-way ANOVA. * Significant differences (*p* < 0.05) between FA− and FA+; # Significant differences (*p* < 0.05) between LPS− and LPS+ by one-way ANOVA. Different letters indicate significant differences by one-way ANOVA. IL6: Interleukin 6; NFκB: Nuclear factor kappa B; TLR2: Toll-like receptor 2; TNFα: Tumor necrosis factor α; COX2: Cyclooxygenase 2.

The effects of FAs and LPS on PBMCs IL6, NFκB, TLR2, TNFα, and COX2 gene expression are shown in Figure 1. LPS significantly increased the expression of IL6, NFκB, TLR2, TNFα, and COX2, although to a different degree depending on the gene. LPS significantly increased the IL6 gene expression in the presence of C16:0, while no changes were observed in the absence of LPS or in the other genes analyzed. The presence of C18:1n9 significantly increased the expression of TLR2, although the presence of LPS mitigated this effect. COX2 gene expression was significantly induced by LPS only in the absence of C18:1n9, as the presence of this fatty acid ameliorated COX2 expression. No significant effects of C18:3n6 were found in any of the analyzed genes. C18:3n3 treatment significantly blocked the activation of the IL6 gene expression induced by LPS, even reaching lower expression values than in the absence of LPS. The NFκB gene expression significantly increased in the presence of C18:3n3. The expression of COX2 was significantly increased by LPS in the absence and in the presence of C18:3n3. The treatment with C20:4n6 prevented the increase of NFκB expression induced by LPS. Similarly to C18:3n3, the presence of C22:6n3 prevented the induction of IL6 gene expression induced by LPS, with values below those observed in the absence of LPS. NFκB gene expression significantly increased in the presence of C22:6n3 or LPS alone, while when combined, C22:6n3 prevented the increase in NFκB. C22:6n3 also mitigated the increased expression of TLR2 and TNFα induced by LPS stimulation.

**Figure 2:**
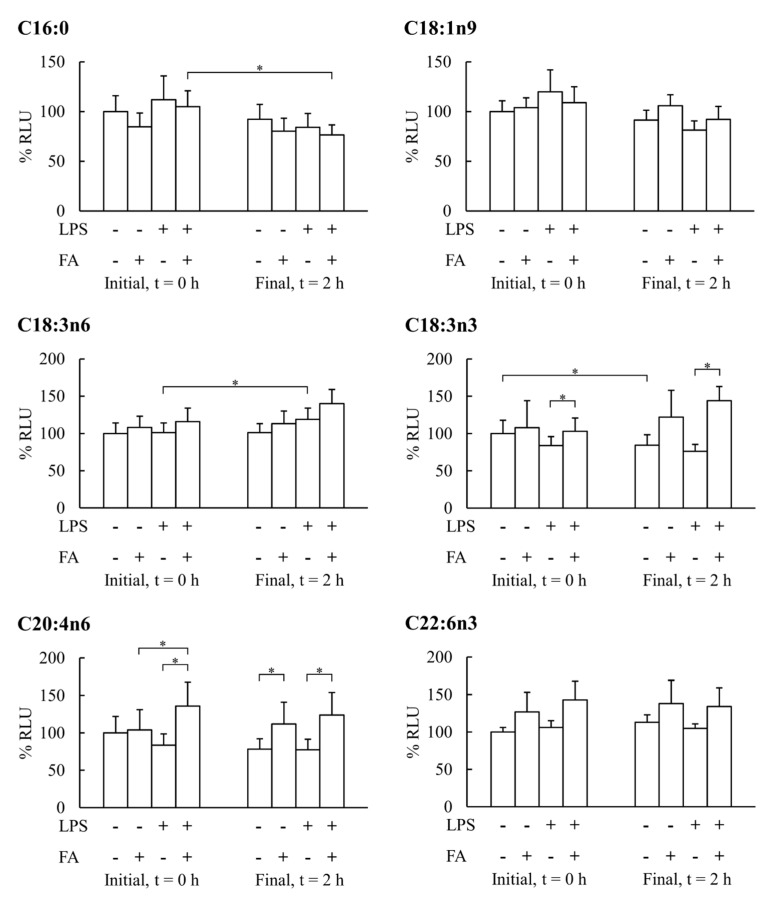
Hydrogen peroxide production by LPS-stimulated PBMCs before and after PBMCs’ incubation with 50 µM of either fatty acid palmitic (C16:0), oleic (C18:1n9), γ-linolenic (C18:3n6), α-linolenic (C18:3n3), arachidonic (C20:4n6), or docosahexaenoic (C22:6n3), or LPS or LPS plus one fatty acid. Statistical analysis: Paired sample *t*-test, *p* < 0.05. * Significant differences (*p* < 0.05) between the groups involved.

The PBMCs’ capabilities to produce H_2_O_2_ after stimulation with LPS, with or without FAs, are shown in Figure 2. The incubation of PBMCs for 2 h in the absence of FAs or LPS maintained the initial H_2_O_2_ production capabilities, although in some cases, there was a slight reduction. The presence of LPS in the PBMCs’ incubation media for 2 h did not affect the initial H_2_O_2_ production capabilities, but in some cases, it increased these capabilities. The combined incubation with C16:0 and LPS significantly decreased H_2_O_2_ production in LPS-stimulated PBMCs, whereas the other FAs did not exert significant effects. C18:3n3 and C20:4n6 increased the LPS-stimulated PBMCs’ H_2_O_2_ production in a similar way before and after incubation for 2 h with these FAs. These effects induced by C18:3n3 and C20:4n6 were enhanced in the presence of LPS in the media.

## 4. Discussion

Dietary lipids modulate the innate and adaptive responses of immune system components [26,27,28,29]. In the present study, we showed that some FAs modulated proinflammatory gene expression in PBMCs and their ability to produce H_2_O_2_ after 2 h stimulation with LPS. The incubation *ex vivo* of PBMCs with oleic, α-linolenic, arachidonic acids, or DHA resulted in different responses, depending on the FA and the gene. Similar regulation by dietary omega-3 FAs in the expression of PBMC genes involved in fatty acid oxidation [21,30], antioxidant genes [31], and inflammatory status [32] have been described in human nutritional studies. In addition, omega-3 FAs can reverse low-grade inflammation in obesity by increasing the synthesis of pro-resolving mediators, such as resolvins, and decreasing proinflammatory mediators [33]. The mechanisms of FA effects on gene expression in PBMCs are not yet known, but it is probable that FAs can directly bind to membrane receptors or change intracellular protein activation [29], modulating TLRs [34] and NFκB [35] signaling pathways and, consequently, the inflammatory response.

NFκB transcription complexes are usually present in the cytoplasm bound to an inhibitor [36]. The activation of NFκB leads to its translocation into the nucleus where it can bind to κB sites and induce the expression of proinflammatory cytokines, antioxidant enzymes, and other genes including IL6, TNFα, and COX2 [16,32,36,37]. NFκB is expressed in almost all cell types and tissues, and specific NFκB binding sites are present in the promoters/enhancers of many genes [36,37]. Inactive NFκB accumulates in the cytoplasm and its activation to bind DNA is independent of de novo protein synthesis; thus, it allows a rapid response to appropriate stimuli. FAs, such as DHA, could interact with the NFκB pathway activation and translocation or affect the binding to κB sites of DNA [17]. The results obtained in the present study show that α-linolenic acid, arachidonic acid, and DHA influence the expression of NFκB and could alter the cellular availability of NFκB and modify its post-translational regulation. Arachidonic acid and DHA prevented the stimulatory effects of LPS treatment on PBMCs. In non-stimulated cells, α-linolenic acid and DHA increased the expression of the NFκB gene and arachidonic acid decreased it.

TNFα and IL6 are considered proinflammatory cytokines when they are released chronically [38]. However, it has been suggested that acute IL6 release may exert anti-inflammatory actions, such as recruitment of mononuclear cells, thought trans-signaling binding to its soluble receptor [39] or inducing the synthesis of anti-inflammatory IL-10 [38]. The inflammatory properties of TNFα lead to the recruitment and activation of immune cells to the injury site and induce the production of various interleukins such IL1, IL6, and IL8 [40]. It has been reported that dietary long-chain omega-3 FAs suppress the synthesis of TNFα, IL1α, and IL1β on mononuclear cells stimulated in vitro with LPS [10,18]. We also showed similar features as DHA and α-linolenic acid prevented the increase of IL6 or TNFα gene expression induced by LPS stimulation of PBMCs. 

Several pathways that finally regulate NFκB signaling are orchestrated by TLRs [13,41,42]. TLRs mediate several processes in the inflammatory cascade upon LPS stimulation, such as the production of TNFα and other inflammatory cytokines, chemokines, and adhesion molecules through activation of NFκB, which leads the immune system to detect infection and react to it [42,43]. In contrast, pathological dysregulation of this process is a hallmark of inflammatory damage, autoimmune diseases, and possibly cancer [44]. It is noticeable that selected free FAs, such as oleic acid and DHA, affect TLR2 gene expression in PBMCs, although with a different pattern. Specifically, oleic acid enhances TLR2 gene expression by non-stimulated PBMCs, whereas DHA diminishes the LPS effect on TLR2 gene expression. Furthermore, it has been reported that dietary DHA can interfere with the TLRs’ activation by LPS [10,45]. PBMCs overexpressed TLR2 during infection [46], and it is important to consider that its modulation could be a double-edged sword, because the inhibition of TLR function can help control the chronic inflammation state, but on the other hand, it could increase susceptibility to infections [47].

COX2 is an inducible isoform of cyclooxygenase involved in the inflammatory response due to its role in prostaglandin synthesis [48]. In a previous study, we observed a potentiating effect of DHA diet supplementation on the levels of COX2 protein in PBMC without changes in plasma prostaglandin levels [11]. DHA can compete with arachidonic acid as a substrate for COX2 reducing the production of inflammatory mediators playing a critical role in the initial inflammatory response [11]. No effects of DHA, neither of palmitic, α-linolenic, γ-linolenic, and arachidonic acids, have been observed on COX2 gene expression in LPS-stimulated or non-stimulated PBMCs. Only oleic acid decreased the COX2 gene expression in PBMCs, with or without LPS stimulation. It points out a possible anti-inflammatory effect of oleic acid decreasing the capacity to produce proinflammatory lipid mediators such as prostaglandins. The effect of oleic acid, which represents 70–80% of olive oil composition, in the immune system is far less investigated than that of polyunsaturated FAs. Previous work showed that oleic acid stimulates lymphocyte proliferation at concentration between 12.5 and 25 µM and decreases it at concentrations greater than 75 µM [49]. The nitroalkene form of oleic, nitrated oleic acid, which is endogenously produced [50], has anti-inflammatory properties and interacts with NFκB [51] and PPARγ [52]. Other monounsaturated FAs such as palmitoleic acid also modulate inflammatory processes [29,53]. Monounsaturated FAs modulate important transcription factors involved in inflammatory pathways, such as NF-kB [35]. In the present study, we showed that oleic acid promotes the overexpression of TLR2 and mitigates the activation of COX2 in PBMCs. Further investigation on oleic acid and derivatives is needed to understand their role on immune status. 

LPS stimulation of PBMCs induces ROS production [54], which, in turn, is involved in TLR-associated activation of NFκB [55]. Dietary supplementation with FAs protects against oxidative damage [31] and enhances antioxidant gene expression in PBMCs induced by acute exercise [31]. FAs could alter the oxidative balance of PBMCs and their immunomodulatory function. In fact, α-linolenic acid or arachidonic acid enhance H_2_O_2_ production by PBMCs stimulated with LPS when measured at time 0, whereas palmitic acid, oleic acid, γ-linolenic acid, and DHA do not have influence. These results show an acute and rapid effect of α-linolenic acid or arachidonic acid on intracellular H_2_O_2_ production measured with the DCFH-DA probe. The increased H_2_O_2_ production by PBMCs after LPS exposure could be a multifactorial response derived from the increased cellular metabolism induced by the activation of TLRs and NFkB signaling pathway, enhancing mitochondrial respiratory chain electron leakage. Mitochondria are the major cellular site for ROS production in PBMCs [56], although another source is the NADPH oxidase, an enzyme localized in the plasma membrane that releases H_2_O_2_ to the extracellular compartment. Incubation of PBMCs for 2 h with or without LPS stimulation maintains their initial H_2_O_2_ production capacity, demonstrating the maintenance of cell viability for this period in accordance with previous experiments [57]. An enhanced production of H_2_O_2_ by α-linolenic or arachidonic acids and the absence of effects by palmitic, oleic acid, γ-linolenic acid, and DHA were observed in LPS-stimulated PBMCs. This is evidence of the specific role of α-linolenic and arachidonic acids in the oxidative response of PBMCs stimulated with LPS. In addition, H_2_O_2_ modulates inflammatory and antioxidant gene expression in ex vivo PBMCs from patients with metabolic syndrome [57], suggesting a possible indirect effect on H_2_O_2_ overproduction by LPS-stimulated PBMCs in the presence of α-linolenic and arachidonic acids.

Equimolar doses of 50 μM of each FA tested were used in order to compare their potential to influence the gene expression and H_2_O_2_ production in PBMCs; however, some concentrations used were not at the plasma physiological range [58,59]. There are few references to individual plasma-free FA expressed in concentration [58,59,60,61], although the plasma concentration of individual FAs is used as a biomarker of some pathologies [60,61]. Free FAs are present in blood at concentrations from 100 µM to 1 mM, and almost all are bound to serum albumin. Considering these reference values [58,59,60,61], the concentration assayed for oleic, γ-linolenic, and arachidonic acids are similar than those in plasma, but the palmitic concentration is lower, and the α-linolenic acid and DHA are higher than plasma levels. In addition, it has been pointed out that only unbound free FAs in plasma are physiologically active [62]; thus, determining the physiological effects of individual free FAs would require the use of nanomolar concentrations. Furthermore, plasma contains a mixture of the different free FAs, and the individual free FAs assayed differently affect proinflammatory gene expression. Consequently, the direct translation of the observed effects of the FAs assayed on the inflammatory and H_2_O_2_ production capabilities to *in vivo* conditions might be difficult in some cases. Despite this, some coincidences were found between the results of diet supplementation with DHA and the ex vivo effects on PBMCs [17,31]. Similarly to the *ex vivo* effects of DHA on PBMCs, the dietary supplementation with a beverage based on almonds and olive oil enriched with α-tocopherol acetate and DHA protected LPS-stimulated PBMCs against inflammation and oxidative stress by influencing the inflammatory, antioxidant gene expression [31] and cytokine release responding to LPS stimulation of PBMCs [17]. The model for studying the inflammatory effects of FAs in human immune cells *ex vivo* could be useful in showing the functional ability of FAs to design functional foods or to evaluate their functional properties.

In summary, depending on FA class, FAs differentially influenced inflammatory gene expression in PBMCs, with or without LPS stimulation. Saturated FAs such as palmitic acid (C16:0) and n-6 polyunsaturated FAs like γ -linolenic acid (C18:3n-6) and arachidonic acid (C20:4n-6) elicited minor effects on inflammatory gene expression in PBMCs, regardless of LPS stimulation, whereas, monounsaturated FAs such as oleic acid (C18:1n9) and n-3 polyunsaturated FAs like α -linolenic acid (C18:3n-3) and DHA (C22:6n-3) reduced inflammatory gene expression. In addition, H_2_O_2_ production in LPS-stimulated PBMCs was potentiated by α -linolenic and arachidonic acids, while palmitic, oleic, and γ -linolenic acids and DHA had no effect. *ex vivo* PBMC assays are easy to perform, rapid, and low-cost methods to assess the effects of FAs or other food bioactives in the design of functional foods.

## Figures and Tables

**Table 1:** Primer sequences and conditions used in real-time PCRs.

Gene	Primer		Conditions	
18S	Fw:	5′-ATGTGAAGTCACTGTGCCAG-3′	95 °C	10 s
	Rv:	5′-GTGTAATCCGTCTCCACAGA-3′	60 °C	10 s
			72 °C	12 s
IL6	Fw:	5′-TACATCCTCGACGGCATCTC-3′	95 °C	10 s
	Rv:	5′-ACTCATCTGCACAGCTCTGG-3′	63 °C	10 s
			72 °C	12 s
NFκβ	Fw:	5′-AAACACTGTGAGGATGGGATCTG-3′	95 °C	10 s
	Rv:	5′-CGAAGCCGACCACCATGT-3′	60 °C	10 s
			72 °C	15 s
TLR2	Fw:	5′-GGGTTGAAGCACTGGACAAT-3′	95 °C	10 s
	Rv:	5′-TTCTTCCTTGGAGAGGCTGA-3′	60 °C	10 s
			72 °C	15 s
TNFα	Fw:	5′-CCCAGGCAGTCAGATCATCTTCTCGGAA-3′	94 °C	10 s
	Rv:	5′-CTGGTTATCTCTCAGCTCCACGCCATT-3′	63 °C	10 s
			72 °C	15 s
COX2	Fw:	5′-TTGCTGGCAGGGTTGCTGGTGGTA-3′	95 °C	10 s
	Rv:	5′-CATCTGCCTGCTCTGGTCAATGGA A-3′	67 °C	10 s
			72 °C	15 s

**Table 2:** Anthropometric, hematological, and biochemical characteristics of participants.

Weight (kg)	92.8 ± 2.8
Height (cm)	165 ± 2
Body Mass Index (BMI, kg/m^2^)	34.0 ± 0.6
Waist circumference (cm)	114 ± 2
Hip circumference (cm)	116 ± 2
Systolic blood pressure (mmHg)	147 ± 3
Diastolic blood pressure (mmHg)	82.5 ± 1.9
Glucose (mg/dL)	127 ± 11
Total cholesterol (mg/dL)	186 ± 8
HDL cholesterol (mg/dL)	41.5 ± 2.2
LDL cholesterol (mg/dL)	110 ± 7
Triglycerides (mg/dL)	185 ± 20
Erythrocytes (10^6^ cells/µL)	4.85 ± 0.11
Haemoglobin (Hb, g/dL)	14.5 ± 0.3
Haematocrit (%)	43.4 ± 0.9
Mean corpuscular volume (MCV, fL)	89.8 ± 1.3
Leukocytes (10^3^ cells/µL)	7.56 ± 0.34
Neutrophils (10^3^ cells/µL)	4.26 ± 0.29
Lymphocytes (10^3^ cells/µL)	2.41 ± 0.16
Monocytes (10^3^ cells/µL)	0.643 ± 0.031
Eosinophils (10^3^ cells/µL)	0.220 ± 0.020
Basophils (10^3^ cells/µL)	0.051 ± 0.005
PBMCs (10^3^ cells/µL)	3.32 ± 0.18
Platelet count (10^3^ cells/µL)	236 ± 10

## Abbreviations

COX2	Cyclooxygenase 2
DAMP	Damage-associated molecular patterns
DHA	Docosahexaenoic acid
FA	Fatty acids
IL	Interleukin
LPS	Lipopolysaccharide
NFκB	Nuclear factor kappa beta
PBMC	Peripheral mononuclear blood cells
PPAR	Peroxisome proliferator activated receptors
ROS	Reactive oxygen species
SPSS	Statistical Package for Social Sciences
TLR	Toll-like receptor
TNFα	Tumor necrosis factor alpha
